# Education and fertility in Egypt: Mediation by women's empowerment

**DOI:** 10.1016/j.ssmph.2019.100488

**Published:** 2019-11-20

**Authors:** Goleen Samari

**Affiliations:** Department of Population & Family Health, Mailman School of Public Health, Columbia University, USA

**Keywords:** Women's empowerment, Women's education, Agency, Fertility, Mediation, Egypt

## Abstract

In 2006, fertility in Egypt reached a two-decade low of 3 births per woman; however, by 2008, the demographic transition reversed, and fertility has remained higher at 3.5 births per woman. Low educational achievement is linked to high fertility. Education is also important in the process of women's empowerment, suggesting that educational achievement lowers fertility through gains in women's agency. However, no studies test this pathway, and evidence on the relationship between education and fertility in Middle Eastern settings is limited. Using longitudinal data from the 2006 and 2012 Egyptian Labor Market Panel Survey (ELMPS), a nationally representative sample of households in Egypt, for 4336 married women aged 15–49 years, this study estimates several linear and mediation regression models of number of births and considers whether women's empowerment explains the relationship between education and number of births. Women's empowerment is operationalized through three measures of instrumental agency: individual household decision making, joint household decision making, and mobility and one measure of intrinsic agency—gender beliefs and attitudes. Higher educational achievement has significant adjusted associations with lower fertility. However, measures of women's agency have mixed mediation associations for education and fertility. Greater individual household decision making and belief in egalitarian gender norms partially mediate the relationship between education and fertility, while greater joint decision making suppresses the relationship. Contrary to expectation, women who have more instrumental agency through more individual and joint household decisions have higher fertility than those who make fewer household decisions. However, women who demonstrate intrinsic agency through greater egalitarian gender beliefs have lower fertility than those who believe in inequitable gender norms. Empowerment programs should focus on improvements in women's education and changing women's intrinsic agency in Egypt, to lower fertility.

## Highlights

• Higher education is associated with lower fertility in Egypt.
• Women who have more instrumental agency have higher fertility.
• Women with intrinsic agency through greater egalitarian beliefs have lower fertility.
• Some measures of agency mediate the relationship between education and fertility.
• Empowerment should be purposefully operationalized and measured.

## 1 Introduction

After a two-decade decline in fertility in Egypt to a low of 3 births per woman in 2006, the demographic transition has reversed and fertility rates have increased (Radovich, El-Shitany, Sholkamy, & Benova, 2018). Between 2011 and 2018, 11 million people were added to the Egyptian population (Karasapan & Shah, 2018). High fertility contributes to rapid population growth and resource scarcity, threatening the health and well-being of Egyptians. Egypt's population of 88 million is projected to grow to 128 million by 2030 (Karasapan & Shah, 2018). The recent rise in fertility in Egypt is an unusual change of direction, as countries that enter fertility transition usually continue to experience lower fertility. High fertility is likely to have grave implications for Egypt by hindering economic development; increasing climate-related challenges; and limiting access to education, employment, food, and potable water, creating health risks for women and children.

High fertility is widely believed to be linked to post-revolution political and social changes, which may have disrupted family planning services or threatened the social status of women, pushing them towards marriage in response to safety concerns (Crandall, VanderEnde, Cheong, Dodell, & Yount, 2016; Samari, 2017b). Evidence from other Middle Eastern countries shows that security threats push women out of education and the labor force and into early marriage and childbearing (Cetorelli, 2014). Research also shows that improvements to women's status through education and employment reduces fertility (Basu, 2002; Hindin, 2000; Jejeebhoy, 1995). However, the pathways and mechanisms linking higher education to lower fertility are poorly understood and have not been empirically tested, particularly in Middle Eastern settings. This study takes a longitudinal approach by using the 2006 and 2012 Egyptian Labor Market Panel Survey (ELMPS) to examine how women's education is associated with fertility over time and whether that association operates through enhanced women's empowerment.

### 1.1 Defining empowerment and education

Women's empowerment is a process that occurs over time and involves *resources* (such as education), *agency* (also referred to as autonomy), and *achievements* (also referred to as outcomes) (Kabeer, 1999). *Resources* include material, human, and social conditions that facilitate the empowerment process by enabling gains in women's agency (Malhotra, Schuler, & Boender, 2005, pp. 71–88). Education is a human resource and a measure of women's status that can promote increases in women's agency and personal and social achievements (Eger, Miller, & Scarles, 2018). Women's *agency* is the ability to define life choices in an evolving historic and social context (Kabeer, 1999). *Agency* includes intrinsic agency (critical thinking skills) and instrumental agency (the ability to formulate one's own strategic choices and to control resources) (Kabeer, 1999; Kim et al., 2007) and provides direct evidence of empowerment. Although there are several related terms for *agency*, including women's status, gender equality, and autonomy, agency is a context-specific, multidimensional construct, operating at individual and collective levels (Richardson, 2018) with application to societies such as Egypt, in which classical patriarchy is still the norm (Kabeer, 2011; Yount, VanderEnde, Dodell, & Cheong, 2016). *Achievements*, or outcomes, are the realization of goals or the result of the empowerment process, including labor market participation or good health (Richardson, 2018).

Education, as a resource, and agency are different parts of the empowerment process, yet education is used improperly, at times, to operationalize empowerment. Although education is considered a key correlate of empowerment, when education alone is used to measure empowerment, researchers cannot disentangle the effects of enabling resources versus the effects of agency (Malhotra et al., 2005, pp. 71–88). At best, education and employment are imprecise proxies for women's agency, since they are not direct measures of critical thinking or decision making. At worst, the failure to separate education and agency prevents researchers from assessing whether improving girls' and women's education and employment—often major elements of programs for girls and women—does in fact change the amount of say they have in their own lives (Balk, 1994; Govindasamy & Malhotra, 1996). Women's education is associated with outcomes such as poverty reduction and economic growth and with greater instrumental and intrinsic agency (Cornwall, 2016; Hanmer & Klugman, 2015; Kabeer, 1999). Researchers are advised to use direct indicators of agency, such as decision making, when possible (Richardson, 2018).

### 1.2 Education and fertility

It is widely recognized that both individual increases in education and average community-level education are associated with lower fertility and longer birth intervals (Axinn & Barber, 2001; Kravdal, 2002; T. C. Martin, 1995; T. C. Martin & Juarez, 1995). However, there is debate as to why education is associated with lower fertility, and there is very limited evidence on how education affects fertility in Middle Eastern and North African countries (Abbasi-Shavazi & Torabi, 2012). There are many mechanisms that can link women's education to their fertility. Education can compete with other resources in women's lives, provide women with the opportunity to learn about modern contraception, or empower women (Mason, 1986).

The link between education and fertility draws on the life course perspective and the idea that women's childbearing decisions and other life decisions are interrelated (Elder, 1998). Because there are competing demands for women's time and resources, and women make choices depending on these constraints, decisions about children are made while taking other life course experiences, such as education, into consideration. Educational attainment may lead to greater participation in the labor force as a competing behavior to having children (S. P. Martin, 2000). The direct impact of socioeconomic variables on the hazard of conception are small, but education and subsequent employment increase the cost of having children, which has a direct influence on fertility outcomes (Balk, 1994; Mason, 1987).

Educational attainment may also increase knowledge of and access to contraceptives, which subsequently lowers fertility (Cleland, 2002, pp. 187–202; T. C.; Martin & Juarez, 1995). Substantial research supports the association between women's educational achievement and increased use of contraceptives (Al Riyami, Afifi, & Mabry, 2004; Jejeebhoy, 1995; Schuler, Hashemi, & Riley, 1997). Other research indicates that the empowerment process is at play, with increases in resources such as education leading to gains in women's agency and more control of fertility behavior, leading to lower fertility (Mason, 1986, 1987). Education creates choices and gives a stronger voice to the vulnerable, like women, in society (Buyinza & Hisali, 2014; Eger et al., 2018). Whether women's empowerment is the pathway by which education influences fertility has not been explored.

### 1.3 Empowerment and fertility

Women's empowerment and household agency is associated with fertility and a wide variety of reproductive outcomes (Prata et al., 2017; Upadhyay et al., 2014). Agency is associated with lower fertility, greater birth spacing, greater contraceptive use, lower ideal family size and fertility preferences, lower risk of unintended pregnancy, and increased access to maternal health care (Prata et al., 2017; Pratley, 2016; Upadhyay et al., 2014).

Theories of how women's agency affects fertility are built around the notion that social institutions of gender, the gender context of the household, and women's socioeconomic position affect women's ability to participate in fertility decision making. Women with agency have influence over interpersonal issues and participate in decision making, including fertility decisions, within the family. Additionally, women who have more control over economic decision making are more likely to be involved in family planning decisions and use a contraceptive method than women who are not empowered (Do & Kurimoto, 2012). In Egypt, women's agency, measured as lack of involvement in decision making and freedom of movement, is associated with women's unmet need for contraception (Al Riyami et al., 2004; Samari, 2018). Greater agency within the household may increase the husband and wife's ability to make joint family decisions related to childbearing.

Education positively contributes to women's agency, helping women have more control in their daily lives (Basu, 2002; Jejeebhoy, 1995; Kabeer, 1999). Women's agency within the household also makes it easier for them to realize the new fertility goals they may have acquired through their years of education (Basu, 2002). Therefore, as women make gains in education, they may also gain household agency and lower their fertility.

### 1.4 Egypt

This study examines the relationships between education, agency, and fertility in Egypt—a country with many commonalities with and differences from South Asian countries where most research on women's empowerment has focused. Egypt is the largest and most densely settled country in the Arab world, characterized by rising fertility, a young population (61% younger than 30 years and 34.2% younger than 15 years), and some recent gains in education but continued limited employment opportunities for women (Assaad & Krafft, 2013; Bertoli & Marchetta, 2015; Karasapan & Shah, 2018; Samari & Pebley, 2018; Yount, Dijkerman, Zureick-Brown, & VanderEnde, 2014).

The educational gender gap in Middle Eastern and North African countries is attributed to stratified gender roles that are still in effect in these settings (Abbasi-Shavazi & Torabi, 2012). In Egypt, women continue to have lower literacy than men (65% literacy for women vs. 82% for men) (USAID, 2017). However, education is becoming more and more accessible to girls in Egypt. The number of primary school students grew by 40% from 2011 to 2016 (Karasapan & Shah, 2018). In 2014, 153,405 girls were not attending school, but in 2017, only 46,695 were not attending school. Equivalent rates of primary school enrollment were observed in 2017 for girls and boys (25.43% for girls and 25.69% for boys) (UNESCO, 2018). Despite the gains in women's education, similar to other countries in the region, women's participation in the labor force remains low, with only 25% of all women ever holding a job (Assaad, Hendy, Lassassi, & Yassin, 2018).

As in South Asia, higher education is not always associated with changes in traditional gender attitudes (Mensch, Ibrahim, Lee, & El-Gibaly, 2003). However, gender-related attitudes seem to be changing: young adults in some parts of Egypt say that women and men should share household decisions (Mensch et al., 2003), and women are making gains in proportions of decisions with spouses (Samari, 2017b). In gender-inequitable contexts, egalitarian beliefs in gender norms are a key aspect of women's intrinsic agency (Yount et al., 2016). In Egypt, families are still organized along patriarchal lines, and married men still occupy the role of head of household (Samari & Pebley, 2018; Yount, 2005a). Later age at first marriage is associated with women's long-term postmarital instrumental agency, including household decision making (Yount, Crandall, & Cheong, 2018). Control, or lack thereof, over household decisions is an important and direct measure of women's agency within Egyptian families (Cheong, Yount, & Crandall, 2017; Samari & Pebley, 2018; Yount et al., 2016, 2018).

Fertility in Egypt is largely marital fertility (less than 1% of births occur outside of marriage), and women on average have three children shortly after marriage (Samari, 2017b). Changes in contraceptive use had the largest impact on the fertility rate prior to 2000, and the fertility rate has remained around 3.5 births per woman since 2008 (Radovich et al., 2018). There is still widespread support for a family model that includes three children (Zalak & Goujon, 2017). Fertility declines in countries in the Middle East and North Africa have been linked to women's educational attainment (Abbasi-Shavazi & Torabi, 2012). However, given the recent gains in women's education, well-educated women in Egypt who lack employment opportunities are having children sooner than expected (Radovich et al., 2018).

More research is needed on how and through what mechanisms women's education affects fertility (Behrman, 2015; Govindasamy & Malhotra, 1996). To date, there are no studies that examine the empowerment process and fertility, or specifically, whether education is associated with fertility through a pathway of women's agency. This understanding is crucial to the promotion of gender equity to facilitate desired demographic outcomes, such as fertility reduction. By examining whether education affects fertility through women's agency in Egypt, this study will contribute to understanding of the mechanisms that link education and fertility.

**Fig. 1:**
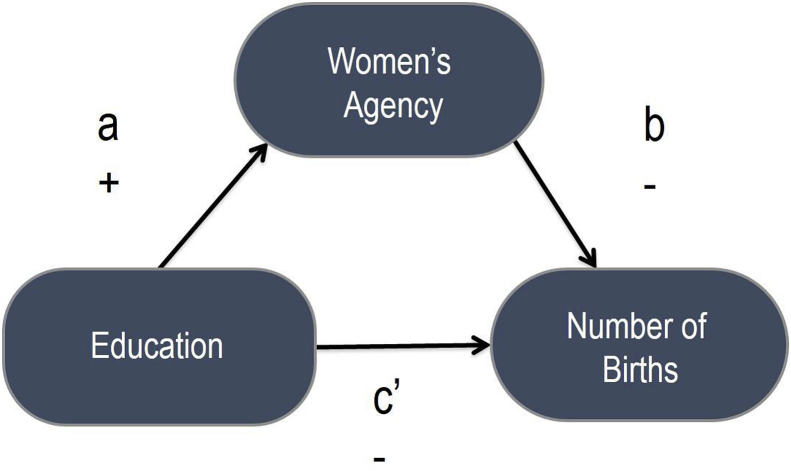
Mediation model of education, agency, and fertility.

Hypotheses include that: (a) higher education is negatively associated with fertility—n other words, more educated women have lower levels of fertility than less educated women; (b) higher education is positively associated with women's instrumental and intrinsic agency; and (c) greater instrumental and intrinsic agency are negatively associated with fertility (Fig. 1).

## 2 Methods

### 2.1 Study design

The Egyptian Labor Market Panel Survey (ELMPS) is a nationally representative panel survey of households in Egypt by the Central Agency for Public Mobilization and Statistics (CAPMAS) and the Economic Research Forum (Assaad & Krafft, 2013). The data include a large nationally representative sample of married women. The data contain individual-level information about education, age, gender, and many other demographic variables as well as household assets and consumption. Egypt is divided into 26 governorates grouped together as the Urban Governorates (Cairo, Alexandria, Port Said, and Suez), Rural Governorates, and the governorates of Upper and Lower Egypt. Lower Egypt is to the north of the Nile Delta, and Upper Egypt is the region south of the Nile Delta. Primary sampling units (PSUs) were allocated to each of the 26 governorates, proportionate to the governorate's size and its urban or rural distribution (Assaad & Krafft, 2013). The 2006 and 2012 waves of the ELMPS include a set of key variables related to agency in Egypt (Cheong et al., 2017; Yount et al., 2016, 2018). All data were self-reported during a face-to-face interview conducted by a trained field interviewer (Assaad & Krafft, 2013). The analytic sample is restricted to married women in their childbearing years (aged 15–49 years) in 2006 who had completed their education by that time and had complete birth histories in 2012 (N = 4336).

### 2.2 Measures

#### 2.2.1 Primary independent variable: education

Education is a continuous variable operationalized as years of education in 2006. Descriptive analyses include a categorical measure of education in which responses were recoded as “0 = No education,” “1 = Primary,” “2 = Secondary,” and “4 = Intermediate or higher.”

#### 2.2.2 Primary dependent variable: fertility

Fertility is a continuous variable of all births reported in a woman's birth history in 2012.

#### 2.2.3 Mediator: Women's agency

Given the classic nature of patriarchy in Egypt, scholars and ethnographic work have demonstrated that salient aspects of agency in Egypt include women's participation in household decisions, freedom of movement in public spaces, and favorable views of egalitarian gender roles (Govindasamy & Malhotra, 1996; Henry, 2011; Kishor, 1995; Sholkamy, 2012; Yount, 2005b; Yount et al., 2016). Thus, women's agency is operationalized with three measures of instrumental agency: individual household decision making, joint household decision making, and mobility and one measure of intrinsic agency—attitudes towards gender norms as reported by female respondents in 2006.

For household decision making, ELMPS respondents were asked to state who in the family had final say on a series of decisions: (a) making large household purchases, (b) making household purchases for daily needs, (c) visits to family, friends or relatives, (d) food that should be cooked each day, (e) getting medical treatment or advice for herself, (f) buying clothes for herself, (g) taking child to the doctor, (h) sending children to school, (i) sending children to school on a daily basis, (j) buying clothes for children. Because the response categories (6 = respondent; 5 = respondent and husband; 4 = respondent, husband, and in-laws; 3 = husband; 2 = in-laws; and 1 = others) do not create an interval, two count variables capture household decision making. A count of the number of times the respondent herself participates in decisions (response category 6), *individual decision making*, is a sum of each time the respondent herself makes a decision and ranges from 0 to 10. A higher count indicates that she makes more household decisions alone. A count of the number of times the respondent and somebody else within the household participate in decisions, *joint decision making*, is a sum of each time the respondent and somebody else (response categories 4 and 5) make a decision. This count also ranges from 0 to 10, with a higher count indicating participation in a greater number of joint household decisions. Test-retest methods demonstrate that *individual and joint decision making* are externally reliable measures of instrumental agency in Egypt (Samari, 2019). Prior work has also examined these measures in relation to fertility and longitudinally in Egypt (Samari, 2017a, b).

*Mobility* is a continuous measure, based on four items in the ELMPS assessing the respondents’ ability to leave the house. Responses were reverse coded (4 = without permission, 3 = just inform them, 2 = need permission, and 1 = cannot go alone) so that higher scores indicated greater control in personal mobility decisions. All items were included, as the principal component analysis indicated that they loaded on one factor and the internal consistency reliability (α = 0.79) was higher with all items included. Items were averaged to create a scale from 0 to 4, with higher values indicating greater freedom of movement. Mobility is coded in similar ways in other studies using the ELMPS (Assaad, Nazier, & Ramadan, 2015; Samari & Pebley, 2018).

*Gender beliefs and attitudes* are a measure of intrinsic agency. In an 11-item continuous scale based on questions in the ELMPS, respondents were asked to consider aspects of women's roles and indicate the extent to which they agreed (1 = strongly disagree, 2 = disagree, 3 = indifferent, 4 = agree, and 5 = strongly agree). Items included: (a) “A woman's place is not only in the household but she should be allowed to work”; (b) “If the wife has a job outside of the house then the husband should help her with the children”; (c) “If the wife has a job outside the house then the husband should help her in household chores”; (d) “A 30-year-old woman who has a good job but is not yet married is to be pitied”; (e) “Girls should go to school to prepare for jobs not just to make them good mothers and wives”; (f) “A woman who has a full-time job cannot be a good mother”; (g) “For a woman's financial autonomy she must work and have earnings”; (h) “Having a full-time job always interferes with a woman's ability to keep a good life with her husband”; (i) “Women should continue to occupy leadership positions in society”; (j) “Boys and girls should get the same amount of schooling”; and (k) “Boys and girls should be treated equally.” Principal component analysis shows that items all load on one factor. Therefore, item scores are averaged creating a scale ranging from 1 for inequitable gender-based attitudes to 5 for very egalitarian attitudes. The items demonstrate internal consistency reliability (α = 0.72). Prior work has highlighted the salience of this measure of egalitarian attitudes in Egypt (Samari, 2017b).

#### 2.2.4 Covariates

Sociodemographic covariates that may be endogenous to education and fertility are accounted for to ensure the sociodemographic variables are not confounding the explanations for primary hypotheses. The woman's mother's education, and her own age at marriage, employment, region of residence, and household wealth were added to the model to check for spuriousness or redundancy. The measure of parental resources is mother's education, a categorical measure in which responses for the mother's educational achievement were recoded as “0 = No education,” “1 = Primary,” and “2 = Secondary or higher.” Age at marriage is a continuous measure of age at first marriage. Ever being employed is a dichotomous variable indicating whether a woman has ever worked for pay. Region is coded as “0 = greater Cairo,” “1 = Alexandria and Suez,” “2 = Urban Lower Egypt,” “3 = Rural Lower Egypt,” “4 = Urban Upper Egypt,” and “5 = Rural Upper Egypt.” Household wealth scores are divided into quintiles: poorest, poor, middle, rich, and richest.

### 2.3 Data analysis

The analysis has two parts. First, descriptive and bivariate associations are examined for all the variables. Second, several multivariate ordinary least squares models examine the association between education, agency, and fertility, while controlling for covariates as mentioned. For the multivariate analyses, education is included and then measures of agency are added to the models. Four separate models estimate the effect of each measure of agency and education on fertility. For sensitivity analyses of temporal ordering, the same models were run predicting women's births between 2006 and 2012. These models produced the same results as fertility in 2012 and, for parsimony, are not included. The separate models of each measure of agency and births by 2012 permit testing of whether any associations between education and fertility are partially mediated by measures of agency. Model diagnostics were performed to assess model fit. Sobel's test (*z*-value = a*b/√[*b*^2^*s_a_^2^ + *a*^2^*s_b_^2^]) and Goodman's test (*z*-value = a*b/√[*b*^2^*s_a_^2^ + *a*^2^*s_b_^2^ - *s*_a_^2^*s_b_^2^]) are used as tests of mediation (MacKinnon, Krull, & Lockwood, 2000). Stata version 15 is used for all analyses (StataCorp. 2016).

## 3 Results

Table 1 shows demographic characteristics of married women in Egypt. The women are between ages 15 and 49 years, and the average age is 30 years. On average, women have between seven and eight years of education. Thirty-seven percent of women have no education, and half the women have received a secondary education or higher. This is in stark contrast to their mothers, a majority of whom have only received a primary education. One-third of women report having ever been employed. Half of the women live in urban areas. In general, respondents have a low amount of personal control in household decisions, with the average score for respondents participating in household decisions equivalent to participating in only two or three decisions (mean = 2.69; SD = 2.54). Respondents also have limited personal control in mobility decisions, with the average score equivalent to a response between “need permission” and “just inform them” (mean = 2.08; SD = 0.73). On average, women reported feeling between indifferent and average to the statements regarding gender norms (mean = 3.74; SD = 0.56).

**Table 1:** Sample characteristics (% or mean [SE]) of married women aged 15–49 years, 2006 and 2012 Egyptian labor market panel survey.

Key Variables	Married Women in 2006 N = 4336	
	N	% or Mean (SD)
**Education (years)**	4336	7.57 (5.63)
**Education**		
None	1605	37.0
Primary	603	13.9
Secondary	1496	34.5
Intermediate or Higher	632	14.6
**Instrumental Agency**		
Individual Household Decision Making	4336	2.69 (2.54)
Joint Household Decision Making	4336	3.37 (2.71)
Mobility	4336	2.08 (0.73)
**Intrinsic Agency**		
Gender Beliefs & Attitudes	4336	3.74 (0.56)
**Current Age (years)**	4336	29.9 (7.17)
**Mother's Education**		
None	35	0.81
Primary	3485	80.4
Secondary or Higher	816	18.8
**Age at First Marriage (years)**	4336	20.5 (4.07)
**Ever Employed**		
No	3058	70.5
Yes	1278	29.5
**Region**		
Greater Cairo	402	9.27
Alexandria & Suez Canal	331	7.63
Urban Lower	509	11.7
Urban Upper	679	15.7
Rural Lower	1319	30.4
Rural Upper	1096	25.3
**Household Wealth Index**		
Poorest	827	19.1
Poorer	970	22.4
Middle	1001	23.1
Richer	857	19.8
Richest	681	15.7
**Births Between 2006 and 2012**	2502	2.97 (1.37)
**Number of Births by 2012**	4336	3.11 (1.41)

**Fig. 2:**
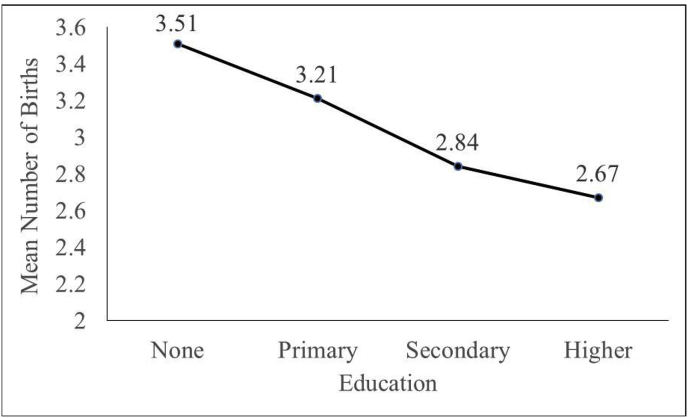
Mean number of births by education.

Fig. 2 shows the mean number of births by educational status. The average number of births decreases with educational achievement. Women with no education have 3.51 births, while women with intermediate or higher education have 2.67 births. Although the average number of births is close to three, the women have anywhere from 0 to 11 births. Fifty-eight percent of the births reported in 2012 occurred after 2006 (2502 births).

Table 2 shows the bivariate ordinary least squares (OLS) regression models of years of education, instrumental and intrinsic agency, and fertility. Model 1 shows a negative statistically significant relationship between education and fertility. For each gain in educational attainment, the number of births lower by .056 (*P* < 0.001). Bivariate models of agency and fertility (Models 2–4) indicate a positive significant relationship between instrumental agency, as measured by individual decision making (*P* < 0.001), joint decision making (*P* < 0.05), and mobility (*P* < 0.001), and number of births. However, intrinsic agency expressed through more egalitarian beliefs in gender norms is associated with fewer births (*P* < 0.001) (Model 5).

**Table 2:** Bivariate ordinary least squares regression models predicting Women's fertility (number of births) in 2012 for married women aged 15–49 years, 2006 and 2012 Egyptian labor market panel survey.

Key Variables in 2006	Model 1		Model 2		Model 3		Model 4		Model 5	
	b	(SE)	b	(SE)	b	(SE)	b	(SE)	b	(SE)
**Education (years)**	−0.056***	(0.00)	–	–	–	–	–	–	–	–
**Instrumental Agency**										
Individual Household Decision Making	–	–	0.05***	(0.01)	–	–	–	–	–	–
Joint Household Decision Making	–	–	–	–	0.02*	(0.01)	–	–	–	–
Mobility	–	–	–	–	–	–	0.26***	(0.03)	–	–
**Intrinsic Agency**										
Gender Beliefs & Attitudes	–	–	–	–	–	–	–	–	−0.24***	(0.04)

Notes: *P < 0.05, **P < 0.01, ***P < 0.001. Standard errors in parentheses.

Table 3 shows the multivariate OLS models of education, agency, and fertility, net of any control variables. Model 1 shows that for each gain in educational attainment, the number of births decreases by 0.029 (*P* < 0.001), all else constant. When the respondent's participation in household decision making is added to the model (see Table 3, Model 2), the effect of education on fertility decreases slightly to 0.028, but it is roughly the same. This relationship remains significant (*P* < 0.001). For each additional household decision women make on their own, they have a higher number of births by 0.06 (*P* < 0.001). With the addition of household decision making, the amount of variance explained by the model increases from 13.1% to 14.1%; however, this small change is significant (*P* < 0.001).

**Table 3:** Multivariate ordinary least squares regression models predicting Women's fertility (number of births) in 2012 for married women aged 15–49 years, 2006 and 2012 Egyptian labor market panel survey (N = 4336).

Key Variables in 2006	Model 1		Model 2		Model 3		Model 4		Model 5	
	b	(SE)	b	(SE)	b	(SE)	b	(SE)	b	(SE)
**Education (years)**	−0.029***	(0.00)	−0.028***	(0.00)	−0.031***	(0.00)	−0.029***	(0.00)	−0.027***	(0.00)
**Instrumental Agency**										
Individual Household Decision Making	–	–	0.06***	(0.01)	–	–	–	–	–	–
Joint Household Decision Making	–	–	–	–	0.04***	(0.01)	–	–	–	–
Mobility	–	–	–	–	–	–	0.30***	(0.03)	–	–
**Intrinsic Agency**										
Gender Beliefs & Attitudes	–	–	–	–	–	–	–	–	−0.10**	(0.04)
**Mother's Education (Ref = Primary)**										
None	0.48*	(0.23)	0.47*	(0.23)	0.49*	(0.23)	0.44	(0.23)	0.47*	(0.23)
Secondary or Higher	0.27	(0.23)	0.27	(0.23)	0.26	(0.23)	0.28	(0.23)	0.26	(0.23)
**Age at Marriage (years)**	−0.09***	(0.01)	−0.09***	(0.01)	−0.09***	(0.01)	−0.09***	(0.01)	−0.09***	(0.01)
**Ever Employed**	0.25***	(0.05)	0.21***	(0.05)	0.25***	(0.05)	0.19***	(0.05)	0.26***	(0.05)
**Region (Ref = Greater Cairo)**										
Alexandria & Suez Canal	0.02	(0.10)	0.05	(0.10)	0.01	(0.10)	0.07	(0.10)	0.01	(0.10)
Urban Lower	0.10	(0.09)	0.08	(0.09)	0.13	(0.09)	0.097	(0.09)	0.09	(0.09)
Urban Upper	0.42***	(0.08)	0.49***	(0.08)	0.43***	(0.08)	0.54***	(0.08)	0.43***	(0.08)
Rural Lower	0.18*	(0.08)	0.18*	(0.08)	0.22**	(0.08)	0.21**	(0.08)	0.18*	(0.08)
Rural Upper	0.40***	(0.08)	0.49***	(0.08)	0.42***	(0.08)	0.55***	(0.08)	0.40***	(0.08)
**Household Wealth Index (Ref = Poorest)**										
Poorer	0.01	(0.06)	0.02	(0.06)	0.01	(0.06)	0.04	(0.06)	0.011	(0.06)
Middle	−0.05	(0.07)	−0.05	(0.07)	−0.06	(0.07)	−0.02	(0.07)	−0.05	(0.07)
Richer	0.11	(0.07)	0.13	(0.07)	0.10	(0.07)	0.15*	(0.07)	0.12	(0.07)
Richest	0.19*	(0.08)	0.20*	(0.08)	0.17*	(0.08)	0.22**	(0.08)	0.19*	(0.08)
**R-squared**	0.131		0.141		0.137		0.153		0.133	
**BIC**	14816.2		14776.4		14793.8		14714.4		14816.7	

Notes: *P < 0.05, **P < 0.01, ***P < 0.001. Standard errors in parentheses.

When the respondent and somebody else's participation in household decision making is added to the model (see Table 3, Model 3), the effect of education on fertility increases to 0.031 and remains significant (*P* < 0.001). For each additional joint household decision women make, they have a higher number of births by 0.04 (*P* < 0.001). With the addition of joint participation in household decision making, the amount of variance explained by the model increases to 13.7% (*P* < 0.001). In Model 4, when mobility is added, the effect of education on fertility does not change (*P* < 0.001).

In Model 5, when gender beliefs and attitudes are added to the model, the effect of education on fertility decreases slightly to 0.027 (*P* < 0.001). Compared to women who believe in inequitable gender norms, women who have more intrinsic agency and believe in more egalitarian gender norms have a lower number of births by 0.10 (*P* < 0.01). For Model 5, the amount of variance explained by the model slightly increases to 13.3% (*P* < 0.001). Model 6 includes all measures of agency to show that each dimension of instrumental and intrinsic agency is uniquely associated with fertility.

Table 4 shows the mediation calculations for education, agency, and fertility. For individual decision making, the indirect effect of education on fertility is 0.001. Because c’ is slightly smaller than c, this shows partial mediation of education on fertility by respondent participation in household decision making. Additionally, the Sobel's test (−2.79; SE = 0.001) is significant (*P* < 0.001), indicating that individual decision making explains about 7% of the association between education and fertility.

**Table 4:** Mediation calculations for ordinary least squares of births in 2012 on Women's education and agency, 2006 and 2012 Egyptian labor market panel survey (N = 4336).

	Instrumental Agency			Intrinsic Agency
	Individual Decision Making	Joint Decision Making	Mobility	Gender Beliefs & Attitudes
**Indirect Effect (c - c')**	0.001	−0.002	0.000	0.002
**Indirect Effect (a x b)**	0.001	0.002	0.001	0.003
**Ratio of Total Effect to Direct Effect (c/c')**	1.036	0.935	1.000	1.074
**Proportion of Total Effect Mediated**	0.070	−0.100	0.000	0.090
**Sobel Test**	−2.79** (0.001)	3.84*** (0.001)	−0.99 (0.001)	−3.54*** (0.001)
**Goodman Test**	−2.81** (0.001)	3.87*** (0.001)	−1.00 (0.001)	−3.55*** (0.001)

Notes: *P < 0.05, **P < 0.01, ***P < 0.001. Standard errors in parentheses.

For joint decision making, the indirect effect of education on fertility is 0.002 (Table 4). Since c’ is bigger than c, this shows that some of the effect of education on fertility is suppressed by joint household decision making (MacKinnon et al., 2000). Additionally, Sobel's test (3.84; SE = 0.001) is significant (*P* < 0.001), indicating that when operationalized as joint participation, agency suppresses the influence of education on fertility. However, because higher education is associated with fewer births, higher education is associated with more joint decision making, and making more joint decisions is associated with a higher number of births, joint decision making inconsistently mediates the relationship between education and fertility. Ten percent of the total effect is inconsistently mediated by instrumental agency, measured as joint decision making.

Mobility does not mediate the effects of education on fertility. For gender beliefs and attitudes, the indirect effect of education on fertility is 0.002 (Table 4). Women's intrinsic agency, measured as gender beliefs and attitudes, partially mediates the relationship between education and fertility (*P* < 0.001). Sobel's test (3.54; SE = 0.001) is significant (*P* < 0.001), and 9% of the total effect is mediated.

## 4 Discussion

This is the first longitudinal study in a Middle Eastern setting to explore whether the mechanism by which education affects fertility is through women's empowerment. The focus on fertility in Egypt is salient, given its rising fertility since 2008 and stalled fertility since 2014 (Radovich et al., 2018). Promotion of gender equality is a long-standing goal of the international development organizations (e.g., the World Bank) because it is positively associated with lower fertility and better health for women and children (Cornwall, 2016; Rao, Vlassoff, & Sarode, 2013). Women's education and agency are critical components of the strategy to promote gender equality, and women's empowerment is a known determinant of lower fertility (Upadhyay et al., 2014). This study extends research on education, gender equality, and fertility by examining whether diverse measures of women's instrumental and intrinsic agency mediate the relationship between education and fertility.

The first hypothesis that higher education would be associated with fewer number of births is supported. Consistent with this hypothesis, fertility is lower for those who have a higher education. This is unsurprising and consistent with the literature that shows lower fertility for women with greater educational achievement (Axinn & Barber, 2001; Basu, 2002; Cleland, 2002, pp. 187–202; Kravdal, 2002; T. C.; Martin, 1995). However, these results show that the relationship between education and fertility is consistent in an important Middle Eastern context. In recent years in Egypt, there are more women with a secondary education than in the past. Furthermore, among women with a secondary or higher education, there is an observed increase in fertility (Radovich et al., 2018). Thus, education is associated with a fewer number of births, although recently in Egypt, higher fertility has been detected at all educational levels than in past years.

The second hypothesis is that women's agency would help explain the mechanism by which education affects fertility. Specifically, higher education would be associated with greater instrumental agency expressed as participation in household decision making, greater mobility, and greater intrinsic agency expressed as more egalitarian attitudes towards gender norms, leading to lower fertility. Higher educational attainment may give women a greater sense of personal control and improved communication skills (Murphy-Graham, 2010), which likely help women negotiate household decisions (Eger et al., 2018). Three of the four measures of agency do help clarify some of the relationship between education and fertility; however, not all measures operate in the same direction. Gender beliefs and attitudes, the measure of intrinsic agency, partially mediate the effects of education on fertility. However, measures of instrumental agency vary in their capacity as mediators. For example, greater individual household decision making partially mediates the relationship between education and fertility, while greater joint decision making suppresses the relationship. The effects of mobility on fertility are completely independent of education, which aligns with research that finds mobility is not associated with resources, such as education, and is primarily associated with subsistence and market employment (Salem, Cheong, & Yount, 2017).

When agency is measured as individual household decision making, the effects of education on fertility are partially mediated. However, this relationship is not in the expected direction. Women with more education have greater instrumental agency and make more individual decisions in the household, and women who make more individual decisions have a higher number of births compared to women who make fewer decisions. Previous studies, primarily in other countries, point to an inverse relationship between agency and fertility (Hindin, 2000; Khan & Raeside, 1997), but recent research from Egypt also shows a positive relationship (Samari, 2017b). Women with more education who demonstrate instrumental agency in Egypt may want more children. Because only a quarter of women participate in the labor market in Egypt (Assaad et al., 2015), educated women who are unemployed are likely largely confined to the home environment (Haghighat, 2013) and may autonomously choose to have children. Furthermore, women's participation in paid work in Egypt is not always associated with greater agency (Salem et al., 2017). In settings like Egypt, where women have limited access to other channels of security, children are of greater value for their mothers' current and future security (Samari, 2017b). Women with more education and agency may formulate their own strategic and instrumental choices in favor of higher fertility.

Joint household decision making suppresses the relationship between education and fertility as does individual decision making; women who made more joint household decisions had a higher number of births than those who made fewer joint decisions. Women who are making joint household decisions may have more of a say in fertility decisions and be making joint decisions in favor of more children. In other settings, preferences for sons over daughters and numbers of siblings influence contraceptive use and overall fertility behavior, creating a complex interplay of economic and sociocultural factors driving fertility (Buyinza & Hisali, 2014). In Egypt, children themselves are found to be empowering for women (Samari, 2017a). There is also widespread indifference between having two or three children, indicating that preferences might not be for fewer children (El-Zeini, 2008). There is a demonstrated preference for sons as well as a preference for having both a son and a daughter (Yount, Langsten, & Hill, 2000). Compared to less educated women, more educated women in Minya, Egypt, report a weaker preference for sons but still prefer them (Yount, 2005b). This could mean that regardless of education driving instrumental agency and participation in decision making, the gender of their children also motivates women's fertility. In Egypt, educated women with experience in paid work are more likely than uneducated women without such experience to express no gender preference in children (Yount, 2005b), indicating that employment also plays a role in fertility decisions. When women have fewer employment opportunities, educated women have less of an incentive to delay marriage and childbearing (Zalak & Goujon, 2017). Additionally, women who are making more joint household decisions may have access to extramarital support from extended kin (Yount et al., 2014). These extended family members, such as mothers-in-law, may also have a say in fertility decisions. Whether women's employment lowers fertility desires in Egypt varies between urban and rural regions depending on how much support from extended kin is available (Zaky, 2004). Future research should consider these and additional mechanisms that help explain the pathways between education, agency, and fertility in Egypt.

Only gender beliefs and attitudes, the measure of intrinsic agency, mediated the relationship between education and fertility in the expected direction. Women that are more educated have egalitarian attitudes towards gender norms, and greater beliefs in egalitarian norms are associated with lower fertility in Egypt. This aligns with research that shows positive gender attitudes are associated with lower fertility (Kaufman, 2000; Samari, 2017b). This also aligns with research that shows more educated women (vs. less educated women) in Egypt have a slightly lower preference for sons (Yount, 2005b). In Egypt, where patriarchal constraints are still very much the norm and community social norms highly influence women's agency (Assaad et al., 2015), women who have more egalitarian beliefs demonstrate a shift in the social normative attitudes towards men and women and sons and daughters. With more education, women make this shift and lower their fertility.

### 4.1 Limitations

Some limitations of this work are notable. First, the ELMPS only includes two waves of data on women's empowerment and fertility, which is an improvement on many cross-sectional surveys of empowerment but limits the longitudinal analyses that can be conducted. Future waves of data will allow for further exploration of mechanisms. There has also been a recent push to measure women's agency as a multidimensional latent construct (Cheong et al., 2017; Miedema, Haardörfer, Girard, & Yount, 2018; Yount et al., 2018). Although the summative scales distinguishing between intrinsic and instrumental agency are an important step in understanding mediation pathways, future exploration of mechanisms that treat agency as a latent construct and use structural equation models are also warranted.

Furthermore, the ELMPS has collected extensive data on work and fertility history, but these data do not include information on women's contraceptive behavior, fertility intentions, or sexual behavior. Having access to this information over time would help clarify the pathways that link education, agency, and fertility over time. Although there were analytic steps considered for temporal ordering between education, agency, and fertility, and sensitivity analyses of births between 2006 and 2012 corroborated the results, some of the births reported in 2012 occurred prior to 2006 when education and agency were measured. These births may have impacted women's agency in 2006. Additionally, existing measures of women's agency provide little contextual information on household dynamics. For example, although separating household decision making into individual and joint decisions contextualizes who is making the decisions, the questions on household decision making provide little insight into discussions women may have had with partners about those household decisions. Men within the household were not administered the questions about women's agency; therefore, the responses are all based on the women's perspective and the discordance between men and women's responses cannot be used to further capture the household power dynamics.

### 4.2 Conclusion

This study takes seriously the call for research on women's empowerment to disentangle the relationship between education, agency, and fertility. This research is the first to consider one mechanism that can explain how education influences fertility in the Egyptian context. Additionally, the ELMPS is a nationally representative, large, longitudinal sample of married women. Although this study only shows associations and does not demonstrate causality, care was taken to enhance temporal ordering issues between education, agency, and fertility by using longitudinal data.

This study also highlights the importance of measuring agency in a purposeful way, as different measures of agency can have different mediation effects. The study also disentangles the effects of individuals versus joint decision making and contextualizes empowerment in Egypt by recognizing that decision making may involve a spouse, which does not necessarily represent a lack of autonomy. Decisions vary by type and not all life choices are created equal (Richardson, 2018), and in many contexts, women can make some decisions alone and other decisions in cooperation with a spouse. At minimum, researchers of empowerment should continue to separately operationalize individual and joint decisions to gain a better understanding of instrumental agency. Findings show that comprehensive measures of women's agency may mediate the relationship between education and fertility but may themselves be associated in complex ways with fertility. This study purposefully considers education and different types of agency as separate constructs and as parts of the empowerment process, to understand their impact on fertility. This study shows that empowerment programs should focus on improvements in women's education and changing gender norms towards women in Egypt to lower fertility.

## Ethics approval statement

This research is exempt. The UCLA Office of Human Research Protection Program determined that the research does not meet the definition of human subject research because the data are available through the Economic Research Forum and are de-identified.
